# A Comprehensive Survey on Intrusion Detection Systems for Healthcare 5.0: Concepts, Challenges, and Practical Applications

**DOI:** 10.3390/s25206261

**Published:** 2025-10-10

**Authors:** Lucas P. Siqueira, Cassio L. Batista, Pedro H. Lui, Juliano F. Kazienko, Silvio E. Quincozes, Vagner E. Quincozes, Daniel Welfer, Shigueo Nomura

**Affiliations:** 1 PPGCC, Universidade Federal de Santa Maria, Santa Maria 97105-900, Brazil; lucas.pittella@acad.ufsm.br (L.P.S.); pedro.lui@redes.ufsm.br (P.H.L.); daniel.welfer@ufsm.br (D.W.); 2 PPGCO, Universidade Federal de Uberlândia, Uberlândia 38408-100, Brazil; cassio.batista@ufu.br (C.L.B.); silvioquincozes@unipampa.edu.br (S.E.Q.); shigueo.nomura@ufu.br (S.N.); 3 PPGES, Universidade Federal do Pampa, Bagé 96460-000, Brazil; 4 PGC, Universidade Federal Fluminense, Niteroi 24040-115, Brazil; vequincozes@midiacom.uff.br

**Keywords:** intrusion detections systems, Healthcare 5.0, survey, Explainable Artificial Intelligence, security, dataset, Internet of Medical Things, practical application

## Abstract

Healthcare 5.0 represents the next evolution in intelligent and interconnected healthcare systems, leveraging emerging technologies such as Artificial Intelligence (AI) and the Internet of Medical Things (IoMT) to enhance patient care and automation. While Intrusion Detection Systems (IDSs) are a critical component for securing these environments, the current literature lacks a systematic analysis that jointly evaluates the effectiveness of AI models, the suitability of datasets, and the role of Explainable Artificial Intelligence (XAI) in the Healthcare 5.0 landscape. To fill this gap, this survey provides a comprehensive review of IDSs for Healthcare 5.0, analyzing state-of-the-art approaches and available datasets. Furthermore, a practical case study is presented, demonstrating that the fusion of network and biomedical features significantly improves threat detection, with physiological signals proving crucial for identifying complex attacks like spoofing. The primary contribution is therefore an integrated analysis that bridges the gap between cybersecurity theory and clinical practice, offering a guide for researchers and practitioners aiming to develop more secure, transparent, and patient-centric systems.

## 1. Introduction

Healthcare has evolved with the advent of Industry 5.0, which emphasizes human-centered approaches and collaboration between people and Cyber–Physical Systems (CPSs). This leads us to the Healthcare 5.0 age, which promotes a structural, behavioral, and cultural transformation, enabling more personalized, secure, and efficient health services for patients [1,2]. A central aspect of Healthcare 5.0 is the transition from a disease-centered approach to a patient-centered model, where the emphasis on prevention—beyond the efficient diagnostics—gains importance [3]. While Healthcare 4.0 integrated CPSs based on cloud or fog architectures with a variety of Artificial Intelligence (AI) algorithms, Healthcare 5.0 builds on these foundations and shifts the focus toward patient wellness, quality of life, and sustainability [2,4].

Given that Healthcare 5.0 applications introduce new challenges in managing the volume, diversity, and sensitivity of patient-related data, cybersecurity concerns have become substantially more prominent, requiring advanced mechanisms to ensure data protection and system resilience [2,5]. In this context, Intrusion Detection Systems (IDSs) emerge as a critical component for continuously monitoring healthcare infrastructures, detecting malicious activities, and enabling responses to cyberattacks, including data alteration, spoofing, denial-of-service, and others [6].

While the use of AI—particularly Machine Learning (ML)—has become a central strategy in the development of IDSs, including within Healthcare 4.0 environments, the transition to Healthcare 5.0 introduces new demands that remain insufficiently explored. This new paradigm not only amplifies the complexity of healthcare infrastructures but also reshapes the nature of the data involved, now including sensitive biomedical streams and contextual information. These changes raise fundamental questions about how AI-based IDSs should adapt to maintain reliability, responsiveness, and trustworthiness in such environments. In particular, the need for Explainable Artificial Intelligence (XAI) becomes more pressing, as clinical settings require not only accurate but also interpretable and justifiable decisions to support security analysts, medical staff, and regulatory compliance [7].

Despite the growing number of works proposing AI-based IDSs, there is a lack of comprehensive studies that systematically examine their applicability, challenges, and limitations within Healthcare 5.0 ecosystems. To the best of our knowledge, the literature does not contain comprehensive studies regarding intrusion detection in Healthcare 5.0 scenarios [8]. Previous works tend to propose single-case secure Healthcare 5.0 architectures—e.g., employing federated learning (FL), blockchain, and IDSs in prototype designs [9,10,11]—but lack a broad, systematic examination of IDSs across Healthcare 5.0 deployments. Other works explore IDSs in Healthcare 5.0 more generally [12,13,14,15,16,17,18,19,20,21], yet omit biomedical-specific considerations and do not address explainability. Recent surveys dedicated to XAI in healthcare [5,22,23,24,25] discuss explainable models over biomedical data, but exclude the security domain and the IDS context entirely—except for [5], which includes a case study but still lacks a focus on IDSs. Therefore, a deeper investigation into how explainability, feature selection, and dataset suitability influence the effectiveness and trust in IDSs deployed in modern healthcare infrastructures is missing.

The main contribution of this survey is to provide a comprehensive review of Healthcare 5.0 intrusion detection by addressing explainability and the role of biomedical data in such a landscape. For that, it covers related concepts, state-of-the-art approaches, and intrusion detection supported by AI. A practical case study with XAI reinforces learned concepts and techniques by indicating the importance of cyber-biomedical data for detecting intrusions in the Healthcare 5.0 scenario. Finally, to further enrich the readers’ and practitioners’ experience, we shed light on open issues in this area.

The additional contributions of this survey are as follows:

- A thorough survey of IDS approaches tailored for Healthcare 5.0 applications.
- An identification and analysis of the existing Healthcare 5.0-aligned datasets employed in intrusion detection, addressing their strengths and limitations.
- A practical case study shed light on XAI’s impact on enhancing IDS’s effectiveness through network and biomedical features. Thereby, we address a key gap in existing AI-driven security solutions for connected healthcare.
- The discussion of open issues and research challenges in the studied area.

This work is structured as follows. Section 2 presents the background for Healthcare 5.0 security. Section 3 reviews related surveys. Section 5 discusses datasets aligned with this context. Section 6 reports a case study comprising an explainable Intrusion Detection System (IDS) for Healthcare 5.0. In Section 7, challenges and open issues are discussed. Finally, Section 8 presents our final remarks and future research directions.

## 2. Healthcare 5.0: Evolution, Enabling Technologies, and Cybersecurity

The growing complexity of healthcare systems, coupled with the rapid evolution of technologies and social structures, has paved the way for a new paradigm known as Healthcare 5.0. This concept reflects a shift toward intelligent, personalized, and human-centered care, aligned with the broader transformations observed in Society 5.0 and Industry 5.0. To understand this movement, this section traces the historical progression of healthcare in parallel with societal and industrial developments (Section 2.1), and explores the enabling technologies that support a secure and responsive healthcare ecosystem (Section 2.2).

### 2.1. From Healthcare 1.0 to 5.0: A Historical and Conceptual Evolution

**Figure 1:**
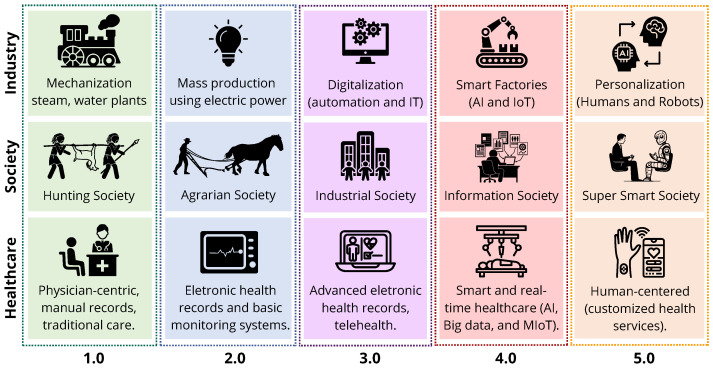
Industry, society, and healthcare evolution concepts.

The evolution of healthcare systems reflects a broader progression in societal and industrial paradigms, moving through five major stages as depicted in Figure 1.

Healthcare 1.0 emerged during the Hunting Society, characterized by physician-centric care, manual records, and traditional face-to-face interactions. Society 2.0 is characterized by the transition to the Agrarian Society. Healthcare 2.0 introduced electronic health records and basic monitoring systems, reflecting the development of more organized and stationary communities. The Industrial Society is marked by the industrial revolution, while the rise of Healthcare 3.0 featured advances in electronic health systems and the emergence of telehealth, aligned with mass production and the widespread use of electric power. Society 4.0 brings the Information Society, while Healthcare 4.0 brought digitalization, smart monitoring, and the integration of AI and Internet of Medical Things (IoMT) into care systems, mirroring the rise in smart factories. Finally, Healthcare 5.0, embedded within the Super Smart Society (Society 5.0), represents a fully personalized, human-centered model where collaboration between intelligent systems and healthcare professionals enables real-time, ethical, and efficient patient care. This trajectory highlights how shifts in social priorities and industrial capabilities have continuously shaped the delivery of healthcare [26,27,28], especially in the last few years, when the world has changed faster. It is important to emphasize that the adoption of these paradigms varies globally; for example, while some regions still operate under Healthcare 3.0 principles, others are already experimenting with 5.0 approaches.

On 22 January 2016, the Government of Japan released the 5th Science and Technology Basic Plan [29]. The plan proposes the idea of “Society 5.0”, a vision of a future society guided by scientific and technological innovation. This term was introduced to define a transformative human-centered future society driven by a new industrial revolution. In this context, the integration of emerging technologies revolutionizes all sectors of society, and especially the healthcare sector [29]. Healthcare 5.0 is not driven solely by technological advancements, but rather by their responsible and human-centric application to improve the patient’s well-being and quality of life [30].

### 2.2. Enabling Technologies

The transition to Healthcare 5.0 has been supported by a technological ecosystem that enables real-time, responsive, and ethically aligned services. While Industry 4.0 emphasized CPSs and large-scale automation, the focus now shifts toward leveraging technology in ways that reinforce social and ecological values, such as trust, personalization, and inclusion [31].

Within this landscape, technologies such as the IoMT, AI, big data, and cloud computing provide the infrastructure necessary to support next-generation healthcare services. These technologies enable advanced sensing, decision support, and intelligent automation, thereby allowing medical environments to become more adaptive and patient-centric [32]. In practical terms, the IoMT spans from wearable devices and in-body sensors to the communication networks and cloud systems that process this information. AI techniques have significantly advanced clinical diagnostics and hospital workflows [24].

Emerging applications like telesurgery and the Tactile Internet (TI) exemplify Healthcare 5.0’s commitment to ultra-reliable, low-latency communication. The TI enables seamless interaction between clinicians, machines, and patients—even at a distance—by providing secure, real-time connectivity. According to the IEEE 1918.1 Working Group, TI allows remote manipulation of physical or virtual objects with human-perceived immediacy [33,34]. Finally, as visualized in Figure 1, the convergence of social, industrial, and healthcare transformations reinforces the unique positioning of Healthcare 5.0. Here, technology acts not only as a driver of efficiency but as a mediator of ethical and personalized care—where patients are not merely treated, but understood, protected, and empowered.

### 2.3. Cybersecurity

Since all Healthcare 5.0 layers handle critical health data, cybersecurity becomes an embedded requirement—not merely a support mechanism. As healthcare environments become increasingly digitized and interconnected, the importance of robust cybersecurity mechanisms grows proportionally. In the context of Healthcare 5.0, where personalized, real-time, and ethically aligned services are prioritized, the protection of sensitive medical data and system integrity is not only a technical requirement but also a social imperative. Techniques such as encryption, secure routing, authentication protocols, and robust back-end infrastructure are required to enhance Confidentiality, Integrity, and Availability (CIA) of healthcare operations and data [35].

Typical IoMT architectures comprise sensing, network, data management, and application layers, each of which presents unique security vulnerabilities. Common attack vectors include Eavesdropping, Denial of Service (DoS), Man-in-the-Middle (MiTM), and Reconnaissance attacks [36]. These threats exploit the open and heterogeneous nature of medical CPSs, making proactive and intelligent threat detection an essential component of any security strategy.

However, under adversarial situations, IDSs are also important to detect violations of these security properties. Among the security solutions, IDSs play a central role in identifying and mitigating abnormal or malicious activities. IDSs are designed to continuously monitor traffic and behavior, detecting deviations from normal patterns that may indicate an ongoing attack. In the healthcare domain, the effectiveness of these systems must go beyond detection accuracy: they must operate within tight latency and reliability constraints while ensuring transparency in their decision-making process [37,38].

AI-based IDSs can be classified into approaches based on ML and Deep Learning (DL). These may employ either centralized training or distributed paradigms, such as FL. ML-based IDSs typically require minimal feature engineering, whereas DL approaches—such as Convolutional Neural Networks (CNNs), Recurrent Neural Networks (RNNs), Long-Short Term Memory Networks (LSTMs), Autoencoders, Generative Adversarial Networks (GANs), and Deep Q-Networks—demand substantial computational resources [39]. FL techniques involve collaborative training across multiple parties to build a global model while preserving data locality and ensuring privacy [40].

IDSs for IoMT can be broadly categorized by their detection methodology. Signature-based IDSs use known patterns and rules to identify threats, but are ineffective against zero-day or unknown attacks. Anomaly-based IDSs, on the other hand, utilize statistical modeling or ML to detect unusual behavior, offering adaptability to emerging threats [41]. However, these approaches often face challenges such as high false positive rates and computational overhead [42].

The integration of XAI into IDS frameworks has gained prominence [43]. Explainability allows stakeholders—such as healthcare providers, system administrators, and regulatory bodies—to understand, trust, and audit the decisions made by detection algorithms [41]. This is especially relevant in environments like Healthcare 5.0, where transparency, accountability, and human-centered design are foundational principles [30]. By incorporating interpretable models or post hoc explanation techniques, IDSs can offer insights into why a specific event was flagged as malicious, thus aligning cybersecurity practices with the ethical and operational values of next-generation healthcare systems [43].

### 2.4. XAI Foundations for IDSs

While IDS solutions have increasingly leveraged ML, DL, and FL, these approaches are predominantly black-box in nature. In contrast, white-box approaches emphasize transparency and human interpretability, aligning with the principles of XAI [40].

In medical contexts, XAI has been increasingly applied to interpret AI-based decisions in critical tasks such as cancer diagnosis [44], ECG signal classification [45], and risk prediction from electronic health records [46]. These applications demonstrate the importance of interpretable models not only for compliance with ethical and regulatory standards but also for fostering clinical trust among healthcare professionals and patients. In scenarios such as anomaly detection in patient monitoring or AI-assisted diagnostics, the ability to explain a prediction can determine whether a recommendation is accepted or rejected in practice [44,47].

Effective XAI systems should satisfy four key criteria [48]: explainability (providing valid justifications for outputs), meaningful understanding (ensuring user comprehension), explanation accuracy (faithfully representing the underlying processes), and knowledge limits (operating within their designed scope). XAI techniques aim to address the opacity of ML, DL, and FL models by providing interpretable and trustworthy explanations. XAI techniques can be categorized by the explanation method, such as feature attribution (e.g., feature maps), textual explanations, and example-based reasoning [49].

**Figure 2:**
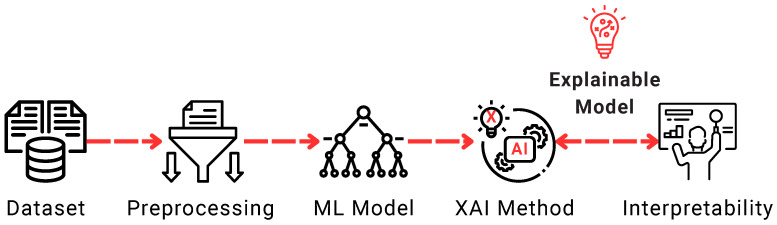
Pipeline of a typical XAI-enhanced ML workflow.

Figure 2 illustrates a typical pipeline for XAI. It begins with the dataset, which undergoes preprocessing to prepare the data for training. An ML model is then trained, such as Gradient Boosting, Naive Bayes, Decision Tree (DT), or K-Nearest Neighbors. After model training, a particular post hoc XAI method—such as Local Interpretable Model-Agnostic Explanations (LIMEs), Shapley Additive Explanations (SHAPs), and Gradient-Based Class Activation Mapping (Grad-CAM). Such methods are presented and discussed in Section 4.2. These explanations enhance the interpretability of the model, providing insights into how decisions are made and allowing end-users to understand and trust the outcomes. This process exemplifies the use of explainability as a complementary stage to model training.

In summary, the background on Healthcare 5.0 security outlines the key concepts of this study. The next section reviews related surveys, highlights existing approaches to intrusion detection and XAI, and sets the stage for identifying research gaps.

## 3. Related Surveys

This section presents the related surveys identified in the literature. Initially, Section 3.1 describes our adopted review protocol and, accordingly, the selected works for our survey. Afterwards, in Section 3.2, reviews regarding intrusion detection in Healthcare 5.0 are examined. Finally, Section 3.3 considers reviews that focus on XAI applied in Healthcare 5.0. In summary, both last sections highlight and discuss the current state of the art and reveal important gaps in the literature.

### 3.1. Literature Review Protocol

**Figure 3:**
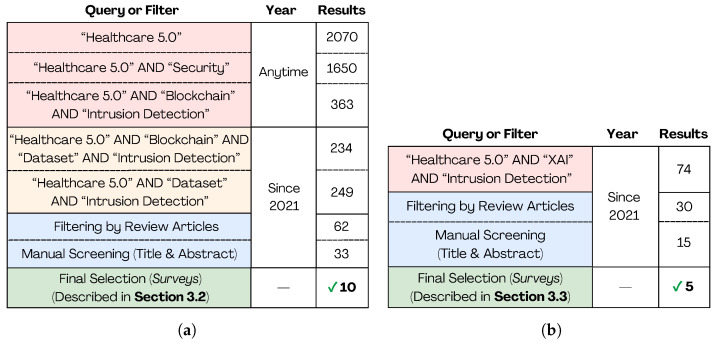
Overview of the filtering and selection process for identifying relevant surveys in the context of Healthcare 5.0. (**a**) Intrusion detection in Healthcare 5.0. (**b**) XAI applied to IDSs in Healthcare 5.0.

The literature review protocol adopted in this study is summarized in Figure 3, which presents the search strategies, filtering steps, and final selection of relevant surveys.

Healthcare 5.0 is a highly challenging topic for research. A search on Google Scholar conducted on 11 March at 17:42 returned 2070 references related to the topic. When narrowing the search to include the theme of security, the number of results decreased to 1650, indicating a more specific research focus. In a new search, including Healthcare 5.0, intrusion detection, and blockchain, 363 results were obtained. Blockchain is gaining importance in IDSs due to the decentralized data storage and privacy protection it affords. However, there are some issues to be resolved in blockchain. FL combined with blockchain has been proposed for building IDSs so as to overcome blockchain issues [11]. Combining the terms Healthcare 5.0, intrusion detection, blockchain, and dataset leads to 234 results in Google Scholar.

Solutions based on AI have challenges for their adoption in the IoMT environment, in which the datasets must work with precision [34]. As IoMT adoption continues to expand, ensuring the robustness of IDS solutions becomes increasingly urgent to protect interconnected healthcare systems from evolving cyber threats. For this proposal, we combined the terms Healthcare 5.0, intrusion detection, and dataset on Google Scholar, which led to 249 results. Further refining, we selected review articles on Google Scholar, resulting in 62 articles found. From these, 33 were selected for the investigation of IDSs and datasets.

The XAI is a technique used to ensure ethical, transparency, responsibility, and accountability for AI applied in healthcare [5,22,38]. Combining the terms Healthcare 5.0, intrusion detection, and XAI leads to 74 results in Google Scholar. Afterwards, the results were filtered by review articles on Google Scholar; from the 30 articles found, 15 were selected. It is worth mentioning that among these selected reviews, no surveys were found that provided a review in a comprehensive manner, combining explainability, IDS, Healthcare 5.0, and practical applications. The literature review protocol is illustrated in Figure 3. It is worth stating that although Intrusion Detection and Prevention Systems (IDPSs) play an important role by additionally taking an action—e.g., host/network quarantine, access control integration, and packet dropping—in order to mitigate the intrusion, they are out of scope of this review. Efficient prevention depends on accurate detection. Hence, the latter remains quite relevant.

Finally, such selected related surveys were classified as (i) AI-based IDS applied to Healthcare 5.0 and (ii) XAI applied to Healthcare 5.0. They are shown in Section 3.2 and Section 3.3 areas separated by a dotted line in Table 1, respectively. Column three represents whether the survey is aligned with Healthcare 5.0 ideas. It is important to reinforce whether the reviews are in the context of Healthcare 5.0. The fourth column shows whether the work addresses intrusion detection. The fifth column shows whether the analyzed review discusses and considers biomedical data and their importance to attain the results. Column 6 identifies those surveys that address XAI and its role in understanding the results. In Column 7, we denote whether the review article presents a practical case study in order to complete and improve the reader’s understanding of the addressed concepts. It is important to point out that some works deal with explainability for disease prediction and monitoring without concerns regarding security. These works are mainly listed in Section 3.3 area from the aforementioned. Also, as shown in Table 1, explainability for intrusion detection in the context of Healthcare 5.0 is still a challenge for researchers and a promising research field. All these works are properly discussed in Section 3.2 and Section 3.3.

### 3.2. Intrusion Detection in Healthcare 5.0

The work [12] provides a comprehensive overview of cybersecurity threats and mitigation strategies for Healthcare and IoT systems using ML, emphasizing practical data collection methods and anomaly detection facilitated by simulators. It systematically categorizes cybersecurity threats across Healthcare and IoT multi-layer architecture and critically analyzes recent attack datasets, feature selection techniques, and mitigation strategies. However, the study does not explore XAI approaches, which could enhance the interpretability and trustworthiness of the proposed ML-based solutions, an increasingly important aspect in safety-critical domains such as healthcare. One reference in the survey pointed to the WUSTL-EHMS-2020 dataset, but with a focus on performance issues.

Three main approaches are used in medical CPS attack detection: anomaly-based (most common), signature-based, and specification-based. Detection systems operate at the device, network, or cloud level, with network-based approaches being predominant [13]. Most studies rely on non-medical contextual data and focus on detecting external threats, malicious insiders, or multiple attack scenarios. Datasets are either public (e.g., TON-IoT, MIMIC III) or privately generated and rarely shared. Notably, few works address the prevention or mitigation of attacks, and XAI techniques are generally absent, limiting the interpretability of detection outcomes.

Research [14] reviews behavior-based intrusion detection in IoMT—primarily anomaly- and specification-based methods—and common prevention and mitigation measures. Although ML is intensely applied to the healthcare landscape, some authors also explore statistical methods, description languages, expert systems, finite state machines, and hybrid approaches to process large data volumes. Performance depends on the chosen datasets (e.g., TON-IoT, NSL-KDD, WUSTL-EHMS-2020, and ECU-IoHT). The research indicates that these datasets do not fully capture the complexity of real-world IoMT environments. Also, it indicates that WUSTL-EHMS-2020 and ECU-IoHT are widely used datasets that incorporate IoMT traffic. Nonetheless, the authors concentrate their efforts on investigating detection performance. They do not consider Healthcare 5.0 issues.

The review [15] presents best practices to promote a secure and resilient smart healthcare ecosystem by synthesizing insights from multidisciplinary perspectives, such as cybersecurity, healthcare management, and sustainability research. IDSs are presented to protect healthcare solutions, IoMT devices, servers, healthcare users, and sensitive patient medical information. However, this work does not explain the impact of biomedical data on its predictive models—specifically, how these insights translate into more patient-centered treatment decisions. Moreover, it devotes relatively little attention to the explainability of its results, which is essential for building trust among clinicians and patients alike.

The overview [16] surveys recent advances in deep-learning-assisted security and privacy frameworks for AI systems, with a focus on safeguarding sensitive health data in IoMT devices. It categorizes DL-based mechanisms for intrusion detection, anomaly identification, and data sharing preserving privacy, and systematically evaluates their methodologies, strengths, and context-specific limitations. The authors conclude by outlining unresolved challenges, such as lightweight architectures and adversarial robustness, to guide future secure and scalable IoMT deployments. However, this survey does not examine the impact of biomedical data on the prediction outcomes (e.g., how these data inform patient-centered treatment). Moreover, it does not address the explainability of the results. Both issues play an important role in clinical trust and adoption.

In [17], the authors conduct a comprehensive survey on personalized healthcare services, in particular of the key requirements of Comprehensive Personalized Healthcare Services (CPHSs) in the modern Healthcare Internet of Things (HIoT), including the definition of personalization and an example use case scenario as a representative for modern HIoT. In their work, the researchers explore a fundamental three-layer architecture for IoT-based healthcare systems using AI-based and non-AI-based approaches. For that, they consider key requirements for CPHS, followed by their strengths and weaknesses in the framework of personalized healthcare services. Highlights different security threats against each layer of the IoT architecture, along with possible AI and non-AI-based solutions. Nevertheless, such research treats IDSs superficially.

The report [18] offers a vision on DL-based IDSs for IoT botnet detection, covering models such as CNNs, RNNs, and GANs, and examining their performance in heterogeneous, resource-constrained settings. By comparing existing work and pinpointing gaps, it delivers actionable insights for designing robust DL-based IDSs and suggests future directions as hybrid ensembles and domain-specific feature selection. In contrast, it does not discuss the impact of biomedical data on the model predictions, neither in theory nor in practice. Also, the explainability of the results is overlooked.

The examination [19] surveys Deep Reinforcement Learning (DRL)-based IDSs in IoT networks from 2014 to 2024, mapping research trends, keyword networks, and publication outlets, and noting a spike in 2022—especially in IEEE and Elsevier venues. It delivers a concise synthesis of DRL approaches, underscores the field’s growing maturity, and pinpoints directions for future work. Notably, the authors do not discuss how biomedical data influence predictive performance (for example, their role in patient-centered treatment). The authors do not provide a practical vision regarding the subject studied.

The review [20] examines ML and DL-based IDSs in IoT environments from 2019 to 2024, with a focus on training paradigms and deployment strategies for resource-constrained edge devices. It synthesizes recent studies to underscore the need for adaptive, cross-domain, low-cost, and energy-efficient IDS solutions, and outlines future directions such as data reduction techniques and FL to boost efficacy across diverse IoT platforms. Its rigorous methodology and comprehensive tables of techniques, datasets, and validation metrics offer a solid foundation for advancing IoT security research and fostering ongoing innovation against evolving cyber threats. It should be noted that this review does not cover the impact of biomedical data on prediction outcomes, considerations of result explainability, or clinical validation.

In [21], recent advances (2020–2024) in ML, DL, and meta-heuristic approaches (e.g., Random Forest (RF), Support Vector Machine (SVM), LSTM, CNN, GAN) for intrusion detection in IoMT applications are examined, emphasizing transparent selection and rigorous evaluation. Key innovations include federated heterogeneous learning frameworks for secure IoMT environments and a unified taxonomy that clarifies existing work and highlights areas for improvement. By synthesizing AI-based IDS technologies tailored to complex medical IoT settings, it lays a solid groundwork for future security research. However, it is worth noting that these studies generally do not explain the impact of biomedical data on predictions (e.g., its role in patient-centered care) and lack clinical validation. Limited attention is given to the explainability of their results.

### 3.3. XAI Applied in Healthcare 5.0

Many healthcare support systems are interested in AI and ML, but face challenges due to a lack of explanation for their decisions. XAI can address the black-box operation of AI models, which have several concerns, including a lack of transparency, explainability, and potential bias in predictions or decisions made in healthcare.

This review [22] explores the development, core principles, and applications for XAI by highlighting its role in boosting transparency, interpretability, and trust across fields, e.g., healthcare, finance, and law. Using the PRISMA framework to analyze 121 articles from 2016 to 2024, it underscores advances in model-agnostic methods such as LIME and SHAP, advocates for user-focused explanation designs, and chronicles evolving regulatory demands for AI accountability. By combining insights from multiple disciplines, it sets the groundwork for responsible AI deployment and collaborative human-AI decision making. However, it should be noted that this review remains largely methodological without illustrating its findings through a practical case study, and it does not address AI-powered IDSs.

The XAI paradigm is increasingly vital in healthcare, where transparency, trust, and interpretability are essential for responsible clinical decision-making. By examining heterogeneous data—from histopathological images to electronic health records—and surveying prevailing machine-learning techniques, the study [23] demonstrates the imperative to move beyond opaque models toward more interpretable solutions. Their proposed XAI framework for healthcare promotes accountability, facilitates clinical validation, and addresses both technical and ethical challenges. By synthesizing multidisciplinary insights and championing the use of open datasets, the work offers clear directions for future investigation. That said, this framework remains predominantly theoretical, without a practical case study to illustrate its real-world applicability, and does not encompass IDSs.

The evaluation [24] review on XAI offers a comprehensive synthesis of XAI methodologies, covering model-agnostic tools like SHAP and LIME, and their applications across domains such as medical imaging and IoT. By categorizing explanation types, models, and functionalities, this work highlights the critical role of XAI in enhancing human decision-support systems, particularly in healthcare, where interpretability fosters trust and clinical relevance. The survey also identifies challenges and future directions, emphasizing the integration of XAI with advanced AI models to improve transparency and accountability. Although insightful, this review overlooks IDSs and lacks a thorough quantitative analysis of how biomedical features influence predictions.

Analyzing 148 studies drawn from 1837 articles across 8 major databases (2014–2024), the review [25] explores frameworks and XAI tools—such as LIME, SHAP, MAPLE, and attention mechanisms—that enhance transparency and interpretability of AI in safety-critical medical contexts. It underscores the value of taxonomies to guide ethical, reliable AI integration for improved patient care, and it emphasizes inherently interpretable models alongside local and global explanation methods. The work systematically organizes current techniques, datasets, and open challenges to advance trustworthy AI adoption in clinical practice. Nevertheless, this work remains primarily conceptual, lacking empirical validation through practical implementations or quantitative evaluations.

This report [5] provides a comprehensive survey and an architectural framework for XAI in Healthcare 5.0, addressing key challenges of interpretability, privacy, and real-time analytics in clinical decision-making. Its main academic contribution lies in an end-to-end XAI-enabled system for medical image classification and segmentation, combining DL with Federated Transfer Learning to protect patient data, demonstrated through a COVID-19 detection case study. The integration of an explainability diagnostic module (XDM) further enhances transparency, trust, and model reliability, supporting broader adoption in healthcare ecosystems. Nonetheless, the study does not explore applications in IDSs, limiting its relevance to cybersecurity-driven healthcare scenarios.

### 3.4. Discussion

In this section, we have discussed related reviews, especially those focused on IDSs and XAI in the context of Healthcare 5.0. Our efforts revealed—as compiled in Table 1—the lack of reviews that consider the importance of biomedical data for intrusion detection purposes. Furthermore, as highlighted by recent surveys [5,12,13,22], significant progress has been made in reviewing IDSs and XAI within the healthcare scenario. Nevertheless, such surveys typically address IDSs and explainability as separate research lines.

Although some reviews seek to show the importance of adopting biomedical data for explainability, there is a lack of exploration of such subjects, as well as IDS landscapes. As our main contribution, we emphasize that this is the first survey to systematically examine explainable Intrusion Detection Systems within the Healthcare 5.0 context, considering both biomedical and network data.

Beyond existing reviews in this research area, the literature has presented relevant methods and directions for building IDSs within Healthcare 5.0. The next section addresses such issues.

## 4. Intrusion Detection Methods and Directions in Healthcare 5.0

This section sheds light on trends and proposed methods targeted to improve intrusion detection in the Healthcare 5.0 landscape. In Section 4.1, we discuss techniques and trends for building IDSs in Healthcare 5.0 scenarios. In Section 4.2, the explainability of the results and techniques to attain it are the target of our examination.

### 4.1. Detection Techniques and Emerging Trends

In the first place, it is important to notice that traditional approaches are quite useful for intrusion detection in Healthcare 5.0, namely anomaly-based detection (the most prevalent), signature-based detection, and specification-based detection. These techniques are implemented across various levels—including device, network, and cloud/cloudlet layers—with a majority of studies adopting network-level detection using non-medical contextual information [13].

Nevertheless, recent advances in AI have introduced a new era for IDSs by employing ML and DL models [50]. These systems demonstrate strong performance in identifying both known and novel threats by learning behavioral patterns from network traffic. Among ML methods, ensemble-based techniques such as EIDS-HS [6] have proven robust by combining multiple classifiers to improve detection accuracy. However, further investigation is required into their performance on specific attack types, such as DDoS in IoT contexts [42].

DL models like CNNs and LSTMs are especially effective in identifying complex anomalies in sequential and grid data [51], while hybrid methods enhance performance by integrating ML, DL, and optimization strategies. Emerging approaches also include adversarial attack resilience [52], rule-based expert systems [53], and 6G-enabled anomaly detection using improved isolation forests and particle swarm optimization [54].

Additionally, to overcome the privacy, scalability, and trust challenges inherent in centralized IDSs, researchers have explored decentralized and collaborative models. FL and blockchain integration are prominent among them.

In [11], the authors propose a federated DL system (AT-DLM) for intrusion and disease detection, ensuring privacy and collaborative learning. Similarly, ref. [55] presents a federated deep extreme learning model with blockchain to secure healthcare analytics, and [56] introduces BFLIDS, which combines FL with blockchain to protect data integrity and reduce reliance on centralized points of failure.

Intelligent IDSs like FIDANN [57] use optimized neural networks (DMO-ANN) trained via FL to achieve privacy-preserving and efficient detection. Other works further incorporate meta-learning and ensemble architectures to enable adaptive and resilient detection [58]. Although FL reduces data transfer and enhances privacy compliance [59], its integration with XAI and distributed optimization is still in early stages and requires further investigation.

### 4.2. Explainability and Model Transparency in IDSs

As AI-based IDSs become more prevalent, explainability has emerged as a critical requirement to foster trust and transparency in clinical environments. XAI techniques allow stakeholders to understand, validate, and trust in model predictions—especially vital in safety-critical domains like healthcare.

Several studies integrate SHAP to interpret model outputs. For example, ref. [60] applies SHAP to ensemble classifiers such as DTs and RFs. A hybrid approach proposed by [61] uses SHAP-based feature selection combined with bagging and boosting to improve model robustness and interpretability. Likewise, studies such as [59,62] employ SHAP in federated environments to maintain consistent interpretability across distributed models.

Despite progress, XAI integration into IDSs remains underexplored in IoT and Healthcare 5.0 environments [63]. There is also a lack of consensus regarding the definition and evaluation of explainability, as highlighted in the review by [64], indicating a need for user-centered formalization and metrics. However, XAI has been pointed out as the solution for trust, informativeness, accountability, causability, and fair and ethical decision-making instrument for white box decision, and is central in Healthcare 5.0 [65]. One of the most crucial concerns in the Healthcare sector is data sensitivity, which is responsible for the life and wellness of the patients. Different stakeholders need different explainability.

There are a variety of techniques for delving into the inner workings of models. However, they frequently come with an accuracy reduction, which affects the prediction performance. Therefore, the choice of interpretability methods in the domain of cybersecurity is limited [66]. According to the survey [22], researchers have adopted the following explainability tools for intrusion detection context: LIME, SHAP, Grad-CAM, Class Activation Mapping (CAM), Permutation Importance (PI), DT, Bayesian Networks, and Federated Transfer Learning.

#### Techniques and Libraries for XAI

In order to delve into our exposition accomplished in Section 2.4, some prominent techniques tailored to provide explainability are properly presented in this section.

LIME represents a model-agnostic local post-hoc approach. It is an open-source library built and designed to interpret decisions by ML models [67]. LIME helps explain instance-based explanations in IDSs, spam filtering, and similar tasks. However, it struggles with high-dimensional data and can be inaccurate [22].

Another well-known XAI library is SHAP. It is built using game theory to explain AI prediction and visualize the origins of decisions. It offers local and global explanations useful for various IDS tasks. SHAP provides local feature importance and transparency in attack detection [22]. Shapley values are a concept from game theory, originally developed as a measure to fairly distribute a reward among a set of players contributing to a certain outcome [68]. Due to the increase in computational complexity of SHAP when the number of features increases, an approximation has been proposed, named KernelSHAP [69]. The kernelSHAP method is used to build the simple explanation model of the actual Autoencoder model. The Autoencoder (AE) is an Unsupervised Artificial Neural Network (ANN) architecture. In general, the Autoencoder is trained on normal/benign data only. Accordingly, they can reconstruct benign data with less reconstruction error, but for attack data, it gives a large reconstruction error and provides a major deviation from the benign data [70]. SHAP is computationally expensive on certain models, such as KNN, but runs fast on trees such as gradient-boosted trees from XGBoost [71].

Tree-based SHAP, named TreeSHAP, has faster performance than KernelSHAP [60]. TreeSHAP provided feature importance to explain model predictions, but had low adoption by analysts, did not improve decision-making efficiency, and was isolated from the primary incident management platform, making it harder for analysts to use during triage [22].

SHAP has a number of explainers. The kernelExplainer method is used to build the explanation models for the actual Autoencoder model in network anomaly detection: deep (based on the DeepLIFT algorithm), gradient, kernel (to estimate SHAP for regression and classification models), linear (to compute the SHAP values for a linear model with independent features), tree (to calculate SHAP values for DT models), and sampling (computes SHAP values by using a random permutation of features). SHAP is a robust method that provides the integration of several methods, such as feature importance, feature dependence, interactions, clustering, and summary plots, all included in a single library. SHAP is a method designed to explain individual predictions by quantifying the contribution of each feature to the predicted outcome [72].

LIME and SHAP belong to a model-agnostic approach, according to the XAI categorization presented in Section 2.4. Model-agnostic techniques are typically post hoc in the sense that they probe trained ML models with different data to generate predictions and then use input–output pairs to extract insights [7]. A combination of LIME and SHAP is proposed to provide explanations and increase the interpretability of a black box model that is part of an IDS solution, which performs intrusion detection on IoT devices [73].

CAM is a model-specific technique [74]. It consists of a visualization tool based on CNNs. Two main categories of CAM techniques are Grad-CAM methods and gradient-free CAM methods [75]. Grad-CAM generates visual heatmaps to explain CNN decisions in malware classification. Nonetheless, it is limited to visually structured data and is not suitable for text data.

Global Attribution Mapping (GAM) offers global explainability for neural network-based NIDS models, but lacks granularity in local decision-making. T-Distributed Stochastic Neighbor Embedding (t-SNE) is used for visualization of high-dimensional data in malware detection, but is difficult to interpret and is not suited for large-scale problems [76]. The GNN explainer explains Graph Neural Networks (GNNs) in cybersecurity applications, which require high computational resources for large graphs. PI, which is a heuristic for correcting biased measures of feature importance. The method normalizes the biased measure based on a permutation test and returns significance P-values for each feature [77]. PI provides global feature importance for IDS models, but it is computationally intensive and can overestimate correlated features. Contextual Importance and Utility (CIU) is model-agnostic and provides uniform explanation concepts for all possible DSS models, ranging from linear models such as the weighted sum, to rule-based systems, DT, fuzzy systems, neural networks, and any ML-based models [78]. CIU provides local context-sensitive explanations, suitable for specific IDS decisions, but can be complex to interpret, especially with multiple interacting features [22].

XAI frameworks act as a tool to explain AI decisions and functioning. It is important in understanding what features contribute to the final decision. One of the most crucial concerns in the Healthcare sector is data sensitivity, which is responsible for the life and wellness of the patients. Different stakeholders need different explainability. XAI is one crucial technology for healthcare 5.0 [65].

The discussion of IDSs and XAI methods highlights the need for reliable datasets to enable effective development and evaluation. Accordingly, the next section reviews datasets for Healthcare 5.0 and assesses their suitability for IDS research.

## 5. Intrusion Detection Datasets for Healthcare 5.0

This section analyzes current IDS datasets, investigating their alignment with Healthcare 5.0. In Section 5.1, the relation among Healthcare 5.0, AI, and datasets is addressed. Section 5.2 presents existing datasets tailored to intrusion detection in the healthcare landscape. To conclude, Section 5.3 summarizes and categorizes previously studied datasets, indicating their alignment with Healthcare 5.0.

### 5.1. Healthcare 5.0, AI, and Datasets

As AI advances, issues regarding its impact on human beings have raised concerns. In Healthcare 5.0, AI should be used adequately to ensure ethical and transparent responsibility. For that, XAI has been proposed as a way of explaining the results. For both model prediction and explainability, datasets are the underlying artifacts that enable the building of the knowledge. A major issue consists of how the literature IDS datasets are aligned to Healthcare 5.0.

It is worth noting that many datasets are targeted to disease identification, without focusing on security, especially intrusion detection. Saraswat et al. [5] present a survey on XAI applications in Healthcare 5.0, addressing interpretability, privacy, and real-time analytics in clinical decision-making for medical image classification. Another study was carried out by Tandel et al. [3]. They propose a machine-based model for a personalized smartwatch-based healthcare solution. In their analysis, they inform that although smartwatch ECG and BP monitoring have shown their effectiveness in monitoring, the diagnosis of hypertension and monitoring the effectiveness of treatment for chronic illnesses is still challenging. Nonetheless, such a study presents no focus on security. Additional medical datasets can be cited, like the MIT-BIH Arrhythmia Database [79]; it provides valuable ECG annotations for arrhythmia research, yet they do not have intrusion detection purposes.

### 5.2. Healthcare IDS Datasets

The CICIoMT2024 dataset is proposed by Dadkhah et al. [80], and it tackles security issues in AI by providing a benchmark for multi-protocol security assessment. Developed using a testbed of 40 IoMT devices (25 real, 15 simulated), including biomedical sensors such as heart rate monitors, oxygen saturation rings, and infusion pumps, the dataset captures network traffic across Wi-Fi, MQTT, and Bluetooth protocols. It incorporates 18 attack scenarios spanning five categories: DDoS, DoS, Recon, MQTT exploitation, and spoofing, enabling robust evaluation of IDSs. Unlike generic IoT datasets, CICIoMT2024 focuses on healthcare-specific threats, combining network metadata with biomedical device behavior to enhance ML-based anomaly detection. Although the dataset supports the Healthcare 5.0 objectives by emphasizing secure and interconnected medical systems, its alignment is partial because it is not composed of network traffic associated with biomedical data generated by biomedical devices.

Ghubaish et al. [81] proposed a Hybrid Deep Reinforcement Learning Intrusion Detection System (HDRL-IDS) to secure 5G medical applications against vulnerabilities in Multi-access Edge Computing (MEC). Their system uses a Deep Deterministic Policy Gradient (DDPG) framework to combine network (NIDS) and host (HIDS) features. Using a custom dataset (WUST-HDRL-2024) generated from an emulated 5G testbed, their experiments demonstrated that the hybrid model significantly outperformed approaches relying on only a single feature source (network or host). By addressing security in 5G and IoMT, the work strongly supports the technological foundation of Healthcare 5.0, which uses these systems for personalized real-time monitoring. Nevertheless, the alignment is incomplete from a security standpoint, as the proposed dataset does not integrate the available biomedical data, a limitation that prevents the creation of truly personalized security measures and thus renders its alignment with the Healthcare 5.0 paradigm partial.

Hady et al. [37] introduced a strong healthcare-centric approach by developing an IDS that integrates both network traffic and real-time biomedical data. To achieve this, they built the Enhanced Healthcare Monitoring System (EHMS) testbed, where they simulated MitM attacks while collecting data, which resulted in the WUTC-EHMS-2020 dataset. Their analysis confirmed the effectiveness of combining these heterogeneous data types for threat detection. However, while methodologically significant, the generalizability of the findings is constrained by the dataset’s relatively small size (16,000 records) and its origin from a controlled testbed rather than a dynamic, real-world clinical environment.

IoT-Flock is an open-source framework for IoT traffic generation proposed by Ghazanfar et al. [82], allowing the creation of emulated use cases, simulation of normal and malicious devices, and generation of IoT traffic. Supports MQTT and CoAP, two application-layer protocols. In another study, Hussain et al. [83] utilized this framework to build a virtual healthcare environment to simulate healthcare devices, such as blood pressure and body temperature sensors, as well as environmental sensors such as CO and air humidity monitors. Malicious and normal traffic was generated from these devices, and a dataset was created using the collected data. The ML classifiers were then applied to detect malicious traffic. The resulting dataset focuses on network and application-layer features but does not include actual biomedical data, despite simulating biomedical sensors like ECG monitors and infusion pumps. While the dataset supports IoMT device security through IDSs and network traffic analysis, its alignment with Healthcare 5.0 is deemed weak due to its reliance on emulated data rather than real-world patient or clinical data.

The ECU-IoHT dataset, introduced by Ahmed et al. [84], addresses a critical gap by providing publicly available network traffic from an Internet of Health Things (IoHT) testbed. Although real biomedical sensors (e.g., temperature, blood pressure) were used in its creation, the dataset exclusively contains network-level flow data rather than the raw physiological measurements. This configuration provides a valuable and reproducible resource for analyzing network attack behaviors, such as DoS and ARP spoofing. While the dataset is instrumental for developing network-level cyber-defenses for IoHT, its alignment with the Healthcare 5.0 paradigm is partial, as the absence of biomedical data prevents the development of security measures personalized to a patient’s physiological state.

To secure Bluetooth-based IoMT devices, Zubair et al. [85] developed the BlueTack dataset and a DL-based IDS. Although generated using real biomedical sensors, the dataset contains only network traffic with emulated payloads, excluding physiological data. The edge-deployed IDS demonstrated strong performance, surpassing traditional ML models. While the approach is partially aligned with the Healthcare 5.0 paradigm through its use of edge and IoMT technologies, the absence of biomedical data limits its ability to deliver human-centered security predictions—an essential component of personalized, patient-driven care.

Areia et al. [36] survey existing ML-based IDS datasets and present IoMT-TrafficData, which consists of two complementary captures: IP-based traffic and Bluetooth traffic. The IP-based capture is divided into packet-level and flow-level records, generated via CoAP and MQTT exchanges in a sports clinic environment equipped with motion sensors and DHT11 modules for temperature and humidity. The Bluetooth capture comprises a WBAN featuring a heart-rate belt and a smartwatch communicating over Bluetooth 4.0 and 5.0, supplemented by an adversarial-attack emulator. However, because the dataset is limited to network-traffic metadata and omits parsed physiological readings (e.g., pulse rate, body temperature), it achieves only partial alignment with Healthcare 5.0 principles.

Zachos et al. [86] present a comprehensive hybrid Anomaly-Based Intrusion Detection System (AIDS) for IoMT networks. A significant limitation is that their dataset is not public. Despite its potential value, the dataset’s restricted accessibility hinders reproducibility and broader adoption. The authors justify its creation due to the lack of existing datasets matching their feature set, yet the decision not to release it limits external validation and comparative studies. This closed-data approach contrasts with the open practices that have accelerated advancements in the field and ultimately represent a weak alignment with the Healthcare 5.0 paradigm, which relies on collaborative, interoperable ecosystems to drive innovation.

The following section discusses and categorizes such datasets based on their alignment with Healthcare 5.0, considering its important properties such as patient-centered care, wellness, real-time monitoring based on medical devices, and the presence of biomedical data.

### 5.3. Discussion

Table 2 summarizes the datasets presented in Section 5.2. It is important to highlight that such a table focuses on IDSs targeted to modern healthcare applications. It emphasizes how these collections bridge network-level threat detection and patient-centered monitoring by compiling diverse data modalities—from raw traffic flows to physiological measurements. Our focus is on evaluating each dataset’s suitability for integrated Healthcare 5.0 environments, where secure connectivity and real-time health tracking must coexist.

The table is organized into eight columns. The first, Dataset, lists the dataset’s name and its respective reference. Soon after, Biomedical Sensor indicates whether data from medical-grade IoT sensors (e.g., wearable ECG or pulse oximeters) are included. The third column, Biomedical Data, signals the presence of vital-sign or physiological measurements in the dataset. The IoT Devices column specifies the device categories (such as IIoMT or IoHT). Afterwards, Network Data confirms the inclusion of network traffic logs. Data Source denotes whether data were collected on real testbeds, emulated environments, or in clinical settings. Availability presents whether the dataset is public or not. Finally, Healthcare 5.0 Alignment rates the adherence of each dataset to patient-centric, wellness-driven principles, ranging from strong (comprehensive sensors, patient-centered, and security integration) to weak.

The alignment levels—weak, partial, and strong—are defined as follows: Datasets categorized as ’weak’ exhibit minimal or indirect relevance to Healthcare 5.0 principles, potentially containing general network traffic or data from IoT devices with minor focus on Healthcare 5.0. ’Partial’ alignment indicates the dataset incorporates some key elements of Healthcare 5.0, such as sensor data or network traffic from healthcare-specific IoT/IoMT devices, but may not fully encompass the comprehensive, integrated, and AI-driven nature of Healthcare 5.0 due to a lack of diverse data sources, patient-specific contexts, or support for proactive security measures. Datasets achieving ’strong’ alignment deeply resonate with Healthcare 5.0’s core tenets, typically featuring rich, multi-modal data from various IoMT devices within a patient-centric framework, often including data relevant to real-time health monitoring, human-centered/biomedical data usage, and the integration of AI-driven insights, making them highly suitable for developing and evaluating IDS solutions tailored for the advanced, interconnected Healthcare 5.0 ecosystem.

After the analysis, the WUSTL-EHMS-2020 dataset [37] stands out as the most critical for advancing Healthcare 5.0 due to its integration of network security and biomedical metrics within a patient-centered framework. Recognizing the absence of IDSs that holistically address both healthcare data integrity and network vulnerabilities, the creators of the EHMS testbed designed a real-time environment where medical sensors transmitted physiological data to a central server via networked devices. Its strong alignment with the Healthcare 5.0 principles, emphasizing real-time IoT integration, patient wellness, and robust security, makes it a benchmark for data sets aiming to secure smart healthcare infrastructures while prioritizing patient-centric outcomes.

While the reviewed datasets provide a foundation for IDS research in Healthcare 5.0, the evaluation of explainable methods requires integrating these datasets into practical case studies. The next section presents a case study demonstrating how XAI techniques can enhance the interpretability and trustworthiness of IDS models, addressing both network and biomedical data.

## 6. Case Study: An Explainable Approach

The integration of XAI into IDSs remains an underexplored but essential frontier in securing Healthcare 5.0 environments. While previous research has demonstrated the benefits of explainability for medical diagnostics and decision support systems [5], its application in cyber defense—particularly in real-time, data-rich healthcare scenarios—has received limited attention.

This section presents a practical case study that investigates how XAI techniques can enhance the interpretability and trustworthiness of AI-based IDS in Healthcare 5.0. The study addresses a critical gap identified in the literature: the lack of models that simultaneously leverage network traffic and biomedical sensor data for threat detection in intelligent medical environments. Although some datasets incorporate elements of medical IoT (e.g., device types or network protocols), few integrate actual physiological data in a manner that enables personalized, patient-aware intrusion detection.

One of the few studies to address this gap is our previous work [87], which introduced a hybrid intrusion detection model combining network and biomedical features using SHAP for global interpretability. Building on that foundation, this case study provides a significant extension through three key contributions: first, a comprehensive scenario-based analysis, where we evaluate both performance and feature importance across network-only, biomedical-only, and combined datasets; second, a deep-dive explainability analysis focused on detecting spoofing attacks using only biomedical signals; and third, the introduction of novel SHAP heatmaps for more granular, instance-level visualization. These enhancements provide a deeper understanding of multi-modal IDS behavior, further aligning with Healthcare 5.0’s demand for secure, interpretable, and patient-centric AI systems.

Using SHAP in a supervised classification pipeline with the WUSTL-EHMS-2020 dataset, this case study demonstrates how combining cyber and biomedical features can enhance both model performance and explainability. This approach aligns with the principles of Healthcare 5.0—namely, security, personalization, and transparency—while also reinforcing the role of XAI as a foundation for ethical and human-centered AI systems [65].

### 6.1. Dataset Description

To evaluate the use of explainable IDS in Healthcare 5.0, we adopted the WUSTL-EHMS-2020 dataset, which was collected in a healthcare-specific simulation environment designed to emulate realistic IoMT scenarios [37]. This testbed integrates both network activity and real-time biomedical sensor readings, offering a unique opportunity to assess the interplay between cyber and physiological features for intrusion detection purposes.

The dataset contains 16,318 samples, each composed of 35 network flow features—such as jitter, packet sizes, and byte counts—and 8 biomedical attributes collected from medical sensors (e.g., heart rate, blood pressure, and oxygen saturation), as detailed in Table 3. Each sample is annotated with two labels: a binary label that classifies it as benign (0) or malicious (1), and a categorical label that specifies the attack type (e.g., spoofing, data alteration), enabling both binary and multiclass classification tasks. Approximately 12.5% of the records correspond to attacks, while the remaining samples are benign.

The threat scenarios in the dataset fall under the MitM category and include [88]:

- Spoofing attacks, in which the attacker passively intercepts packets between the gateway and the server by impersonating a legitimate network device. This compromises data confidentiality by exposing sensitive patient information.
- Data injection attacks, in which the attacker actively modifies the intercepted packets in transit, potentially introducing false medical readings or control commands, thereby violating data integrity and putting patient safety at risk.

All data were captured using the Audit Record Generation and Utilization System (ARGUS), a network monitoring tool designed to log real-time connection records and traffic metadata. This enabled the authors to record both communication-level and physiological signals in a synchronized manner, enhancing the dataset’s fidelity and relevance.

The hybrid nature of WUSTL-EHMS-2020—merging cybersecurity logs with patient biomedical data—makes it highly aligned with Healthcare 5.0 principles. It supports the development of IDS models that are not only accurate but also interpretable, context-aware, and patient-centric. This multi-dimensional perspective helps bridge the gap between technical performance and clinical applicability in modern intrusion detection research.

### 6.2. Methodology

**Figure 4:**
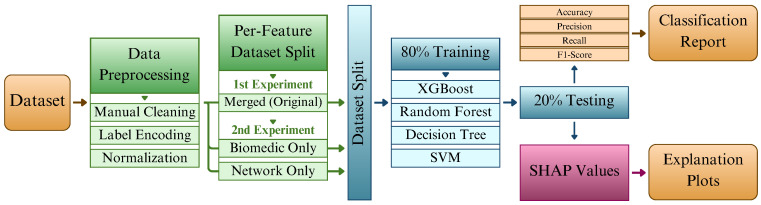
Methodology flowchart.

The methodological steps adopted in this case study are summarized in Figure 4. The process begins with data preprocessing, which involves the removal of features that could introduce bias or have unclear semantics. For instance, the Source MAC Address feature was excluded because the testbed used fixed machines to simulate normal and attack traffic, which would allow the model to trivially learn this distinction. Similarly, the binary Label was removed to prevent the classifier from memorizing the ground truth.

In addition, the Dir and Flgs features were discarded due to the absence of clear documentation in the original dataset. Entries in the Source Port field containing non-numeric values were also eliminated, resulting in a final dataset with 16,315 samples. To ensure consistent labeling, the class originally marked as “normal” in the Category Label field was renamed to “benign”.

Categorical features were encoded using the LabelEncoder class from the scikit-learn library, available on [89], and all numerical features were standardized using StandardScaler. The Source Port feature, although numeric, was also label-encoded to ensure it could be correctly processed in the SHAP-based explainability stage, as port numbers lack meaningful ordinal relationships. Table 3 presents the complete list of features, their types, descriptions, and whether they were included or removed during preprocessing.

Following preprocessing, the WUSTL-EHMS-2020 dataset was employed in two complementary experiments that differed in the feature groups considered. The first experiment (Section 6.3 and Section 6.4) utilized the complete feature set comprising both network and biomedical attributes. This configuration was designated as the merged (original) dataset, given its integration of both data modalities. The second experiment, referred to as the scenario-based XAI analysis (Section 6.5), used the merged dataset as a baseline and derived two additional subsets: one containing only biomedical features and another restricted to network features. As a result, three datasets were analyzed—merged, biomedical-only, and network-only—enabling a comparative assessment of feature group contributions. All datasets were partitioned into 80% for training and 20% for testing, serving as the basis for model development and subsequent explainability analysis.

After preparing the data, we proceeded to the ML phase. Four classifiers were selected for training and evaluation: DT, RF, Support Vector Classifier (SVC), and XGBoost (XGB). The preprocessed dataset was split into training and testing subsets. As illustrated in the flowchart, the classification performance of each model was assessed using evaluation metrics, detailed in the next subsection. The best-performing model was further analyzed using the SHAP library to produce visual explanation plots based on Shapley values, enabling feature-level interpretability of predictions.

### 6.3. Classification Results and Comparison

To evaluate the performance of the trained classifiers, we used four ML algorithms previously described: DT, RF, SVC, and XGB. Each model was trained and tested using a stratified split of the preprocessed dataset, maintaining the original class distribution.

The effectiveness of each model was measured using four standard classification metrics: Accuracy, Recall, Precision, and F1-Score. Accuracy reflects the overall proportion of correct predictions; Recall measures the model’s ability to correctly identify attack samples (true positives); Precision quantifies the reliability of positive classifications by indicating how many of them are actual attacks; and the F1-Score, defined as the harmonic mean between Precision and Recall, provides a balanced view particularly suitable for imbalanced datasets [90]. The formulas for these metrics are presented in Equations (1) to (4):

(1) $$\mathrm{Accuracy}=\frac{TP+TN}{TP+FP+TN+FN}$$

(2) $$\mathrm{Recall}=\frac{TP}{TP+FN}$$

(3) $$\mathrm{Precision}=\frac{TP}{TP+FP}$$

(4) $$F1\mathrm{Score}=2\times \frac{\mathrm{Precision}\times \mathrm{Recall}}{\mathrm{Precision}+\mathrm{Recall}}$$

**Figure 5:**
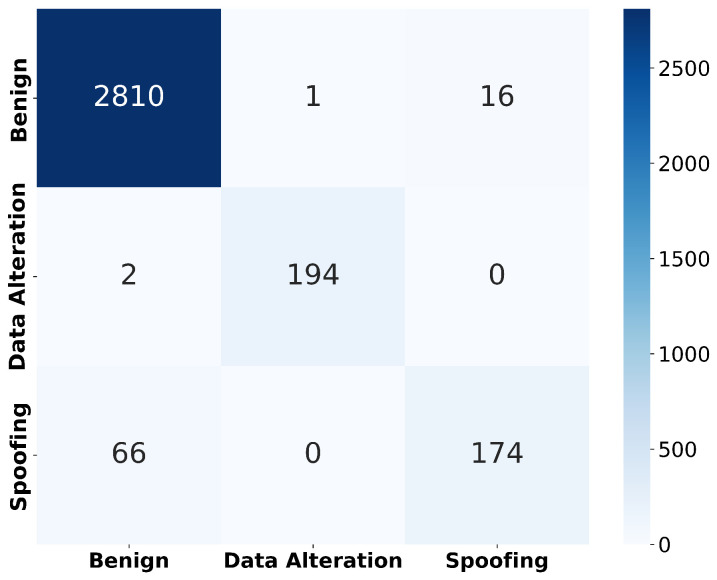
Confusion matrix.

In these expressions, TP, TN, FP, and FN represent the number of true positives, true negatives, false positives, and false negatives, respectively. It is worth mentioning that they can be observed in Figure 5, and they support not only the above expression definitions but can effectively be used to define new metrics, e.g., AUC-ROC [37], allowing us to extend both the results and the analysis.

While Accuracy is a useful global measure, it can be misleading in scenarios with class imbalance, as previously discussed. In the WUSTL-EHMS-2020 dataset, only 12.5% of the instances are attacks, meaning that a model predicting mostly benign samples could still achieve deceptively high accuracy. For this reason, we emphasize the F1-Score as the primary evaluation metric, as it better reflects the classifier’s ability to detect intrusions without being skewed by the prevalence of benign traffic [90].

**Figure 6:**
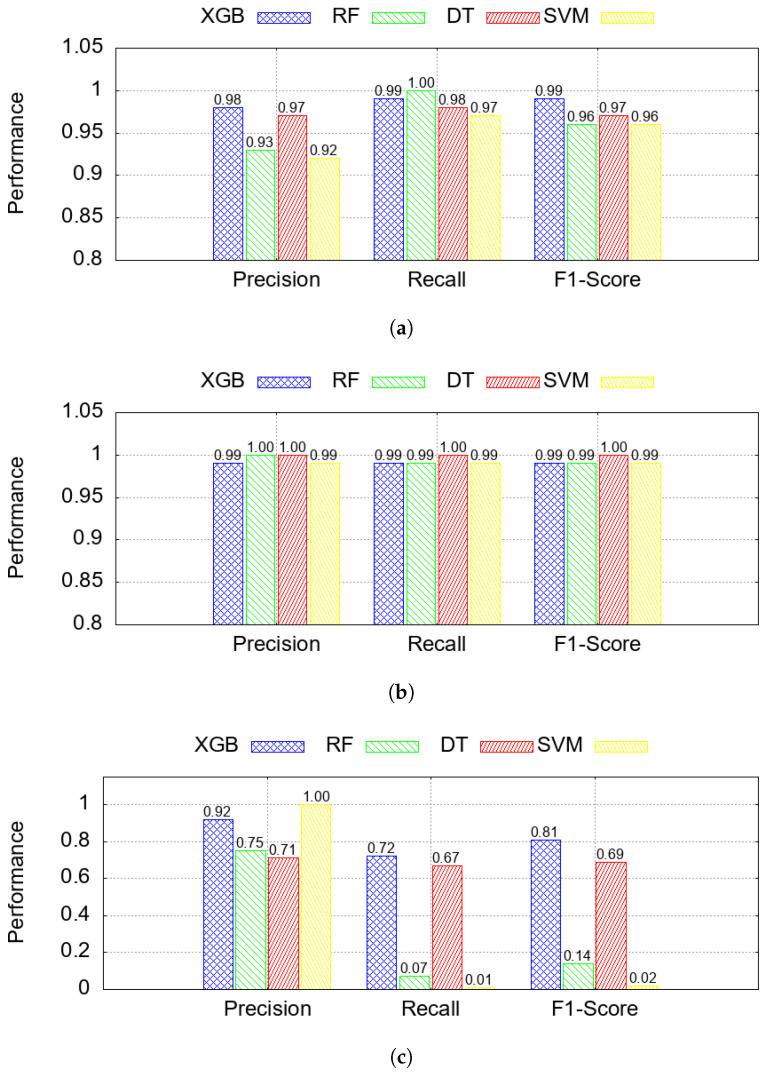
Performance results of different classifier models. (**a**) Benign label. (**b**) Data alteration label. (**c**) Spoofing label.

The classification results are illustrated in Figure 6a–c, which show model performance for each of the three class labels: benign, data alteration, and spoofing. While the four classifiers performed similarly on benign and data alteration samples, XGB generally achieved higher overall performance and was therefore selected for the SHAP-based interpretability analysis presented in the next section [87].

XGB was chosen for its gradient-boosting efficiency and regularization to prevent overfitting, while RF leveraged ensemble bagging and feature randomness for robust generalization. SVC addressed nonlinear decision boundaries in high-dimensional spaces to detect subtle attack patterns, and DT provided an interpretable, rule-based baseline.

Figure 5 shows the confusion matrix from the XGB, where true positives, false negatives, and other classification outcomes are reported. The results indicate that the model performed strongly in distinguishing benign and data alteration instances, with minimal misclassifications, while spoofing detection remained the most challenging case.

### 6.4. SHAP Heatmaps: Local Feature Explanations

**Figure 7:**
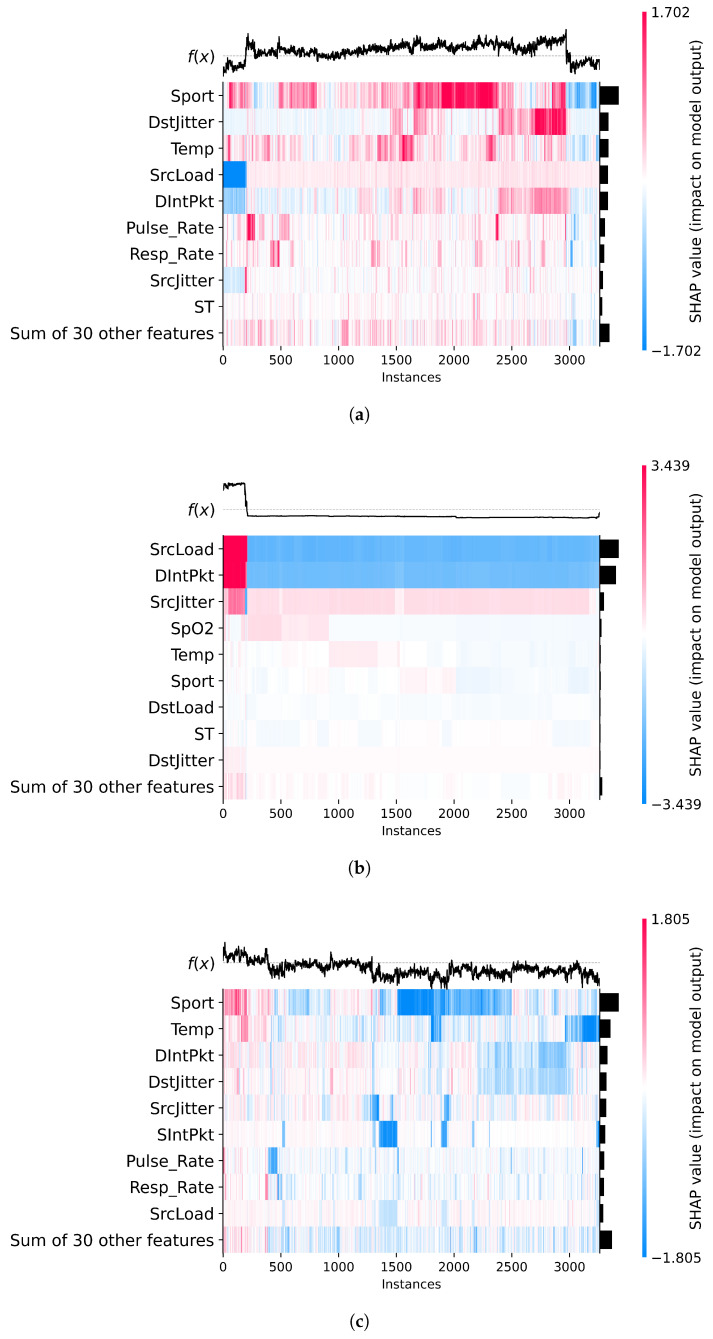
SHAP heatmap plots for different attack types. (**a**) Benign heatmap. (**b**) Data alteration heatmap. (**c**) Spoofing heatmap.

This section presents SHAP heatmap visualizations (Figure 7) that illustrate how individual features contributed to the classification decisions made by the XGB model across different target labels. Unlike plots that display raw values, SHAP heatmaps visualize each feature’s contribution (positive or negative) to the model output on a per-instance basis. Red regions indicate a positive contribution toward the predicted class, while blue indicates a negative one. The horizontal axis represents individual samples, and the vertical axis lists the top contributing features. The black bars on the right show the mean absolute SHAP values across all samples, serving as an indicator of overall feature importance. The f(x) curve above each heatmap represents the model’s raw output score before applying the classification threshold.

In the heatmap corresponding to the benign class (Figure 7a), we observe a complex interplay of both network and biomedical features influencing the model’s decisions. Notably, Sport shows dominant red zones across numerous instances, especially beyond the midpoint of the dataset, indicating a strong and consistent contribution toward benign predictions in certain traffic patterns. Conversely, features such as SrcLoad and DIntPkt display concentrated blue regions, particularly in early instances, suggesting that high traffic load or irregular inter-packet intervals tend to reduce the likelihood of a benign classification. Additionally, biomedical features like Pulse_Rate and Resp_Rate exhibit moderate red contributions and high average SHAP values, highlighting their relevance in characterizing normal operational states. The presence of both blue and red zones across many features reflects the diversity of contexts in which benign traffic appears, with some feature values increasing confidence in benign behavior while others, under specific conditions, signal deviation. This variation underscores the model’s capacity to adapt its reasoning based on nuanced, multivariate patterns.

In the heatmap associated with the data alteration label (Figure 7b), the SHAP values are strikingly concentrated in a small subset of features. SrcLoad and DIntPkt stand out as dominant contributors, exhibiting consistently negative SHAP values (in blue) across nearly all instances. This suggests that lower inter-packet intervals or specific patterns in source-side load are strong indicators of altered data, reducing the model’s confidence in a benign classification. The pronounced black bars on the right confirm the high mean SHAP values for these features, underscoring their pivotal role in the model’s decision-making for this class. Additionally, SrcJitter and the biomedical signal SpO2 show mild yet noticeable influence, indicating that anomalies in packet timing and oxygen saturation may also play a role in identifying this type of attack. In contrast, most other features exert minimal effect, making the interpretability landscape for data alteration considerably more focused and deterministic than that of the benign class.

In the heatmap corresponding to the spoofing class (Figure 7c), a more distributed influence of features is observed when compared to the sharply focused pattern seen in data alteration. Several variables contribute meaningfully to the model’s prediction, including Sport, Temp, DIntPkt, SrcJitter, and SrcLoad, as evidenced by the alternating bands of red and blue along the instances. Notably, Sport and Temp often display positive SHAP values (in red), which may reflect the presence of typical patterns in benign traffic that also appear in spoofed attacks. In contrast, features such as SrcLoad and DIntPkt tend to push the prediction away from a benign label, suggesting their effectiveness in capturing spoofing-specific anomalies. The moderate influence of biomedical signals like Pulse_Rate and Resp_Rate also hints at subtle physiological deviations during spoofing scenarios. Overall, the f(x) plot remains relatively stable, and the presence of multiple significant SHAP bars indicates that this attack category is modeled using a more balanced feature set, underscoring the complexity and variability of spoofing behavior.

Taken together, the heatmaps reveal that the model leverages both network-related and biomedical features to support its predictions. While variables like SrcLoad and DIntPkt often dominate in importance, physiological signals such as Temp, Pulse_Rate, and Resp_Rate appear consistently across different classes. This highlights the relevance of integrating biometric context into intrusion detection—an approach aligned with the principles of Healthcare 5.0, where securing CPSs requires awareness of both digital and human-centered data.

To deepen this interpretability analysis and assess the influence of data modality, we further conducted a scenario-based evaluation using SHAP across biometric-only, network-only, and combined settings, as detailed in Section 6.5.

### 6.5. Scenario-Based XAI Analysis

Building upon the previous analyses, this section explores how feature relevance varies across three operational settings using SHAP: (1) biometric data only, (2) network data only, and (3) a merged dataset combining both modalities. This comparative scenario-based analysis offers a deeper understanding of the isolated and combined influence of physiological and network-level signals on IDS performance within Healthcare 5.0 environments.

**Figure 8:**
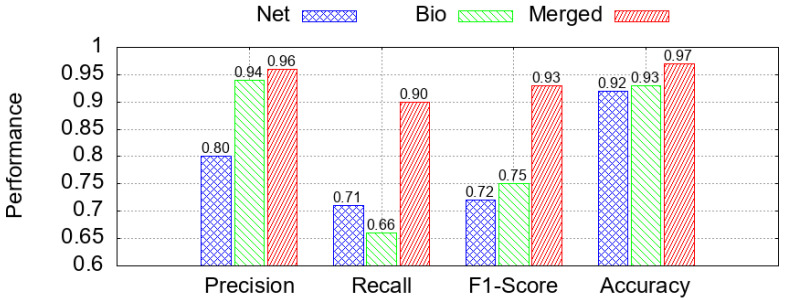
Different metric scenarios.

Figure 8 presents the comparative performance of the three scenarios across four evaluation metrics: Precision, Recall, F1-Score, and Accuracy. The merged dataset consistently outperforms the unimodal setups, especially in Recall and F1-Score, indicating superior ability to identify intrusions while maintaining balanced predictions. While biometric features alone yield high Precision and Accuracy, their limited Recall suggests they miss some attacks when used in isolation. Conversely, the network-only model underperforms across all metrics, highlighting the limited expressiveness of traditional traffic features in complex Healthcare 5.0 environments. These results reinforce the advantage of multimodal IDSs and justify the interpretability analyses that follow.

**Figure 9:**
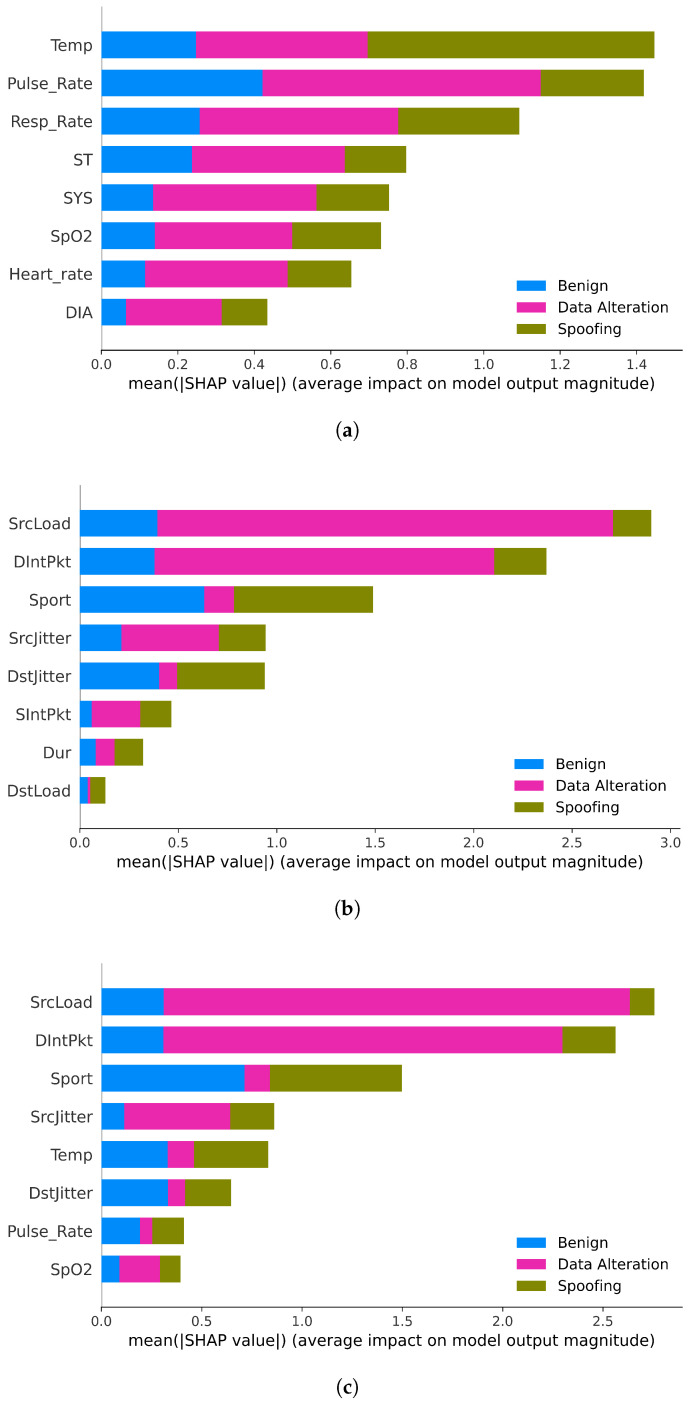
Performance results of different scenarios. (**a**) Bio-only summary bar plot. (**b**) Net-only summary bar plot. (**c**) Merged summary bar plot.

To understand the relative importance of features in each scenario, SHAP summary bar plots were generated for the biometric-only, network-only, and merged datasets (Figure 9a, Figure 9b, and Figure 9c, respectively). Each plot ranks the top features according to their mean absolute SHAP values, representing the average contribution of each variable to the model’s predictions across all instances. These visualizations allow the identification of the most influential attributes in each modality and provide a comparative perspective on how different data types contribute to intrusion detection.

The SHAP summary plots in Figure 9 offer insights into how distinct features contribute to detecting different attack types across the biometric-only, network-only, and merged scenarios. In the biometric-only configuration (Figure 9a), the feature Temp stands out with a mean SHAP value above 1.4 for the detection of spoofing, confirming its relevance as a physiological indicator of biometric tampering. However, several other features, such as Pulse_Rate, Resp_Rate, and ST, exhibit substantial contribution to data alteration detection, as indicated by their pink segments. This suggests that while thermal anomalies are the strongest signal for spoofing, variations in cardiovascular and respiratory metrics are more associated with integrity breaches in biometric data streams.

In contrast, the network-only model (Figure 9b) displays a distinct pattern of discriminative features. SrcLoad and DIntPkt dominate the detection of data alteration, both with mean SHAP values close to 3.0. Meanwhile, Sport, SrcJitter, and DstJitter show greater impact for identifying spoofing, reinforcing the relevance of packet flow irregularities and port manipulation in this attack type. In the merged configuration (Figure 9c), network features such as SrcLoad and DIntPkt remain predominant, but biometric features like Temp, Pulse_Rate, and SpO2 re-emerge among the top contributors. This demonstrates that hybrid models capture cross-domain patterns more effectively, improving detection robustness while aligning with Healthcare 5.0’s emphasis on transparency, accuracy, and contextual intelligence in cyber–physical security systems.

**Figure 10:**
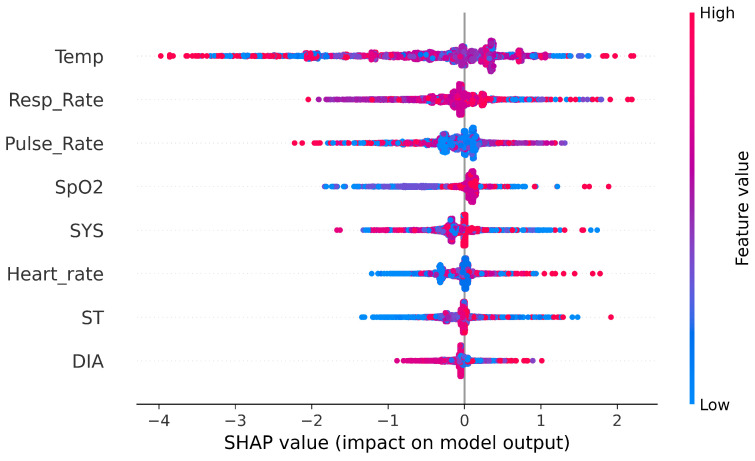
Summary plot of the biomedical subset with a spoofing attack label.

Figure 10 presents the SHAP summary dot plot for the spoofing attack category using only biomedical features. The results confirm that core body temperature and respiratory rate are the most influential predictors: elevated values of both features (represented by magenta points on the right) drive the model toward a spoofing classification, while lower values tend to reduce this likelihood. Pulse rate displays a nuanced behavior, with both low and high values impacting the model, though a slight bias toward positive SHAP values suggests that elevated pulse rates are often—but not always—interpreted as indicative of spoofing. Features like oxygen saturation, systolic pressure, and heart rate variability show intermediate and context-dependent effects, while surface temperature and diastolic pressure contribute minimally, as reflected in their tightly centered SHAP distributions. Altogether, the model detects spoofed signals primarily through exaggerated thermal and respiratory anomalies, while other vital signs play more auxiliary or situational roles.

### 6.6. Discussion

Our case study demonstrated that the fusion of network flow metrics with patient biomedical data yields substantial gains in detection performance. In addition, XAI techniques were applied to uncover why these improvements occur in Healthcare 5.0 environments. By leveraging SHAP across three scenarios (biometrics-only, network-only, and merged), the survey found that physiological signals—particularly body temperature and respiration rate—often dominate spoofing detection, even outperforming traditional network indicators in certain contexts. It also confirmed that hybrid models not only enhance accuracy but also provide critical transparency, revealing, for example, that elevated temperature spikes can be early warning signs of spoofing attempts. The case study illustrates the potential of XAI to improve IDSs’ interpretability in Healthcare 5.0, yet it also highlights limitations and areas that need more investigation.

Despite its advantages, the WUSTL-EHMS-2020 [37] dataset has certain limitations. Its size (16,318 samples) is relatively modest compared to large-scale intrusion detection datasets, which may affect the robustness of training and evaluation. Moreover, because the data were generated in a controlled testbed in a laboratory, they may not fully capture the diversity of real-world Healthcare 5.0 ecosystems, where device types, network infrastructures, and patient populations vary significantly. This introduces potential biases, particularly due to the predominance of generated traffic and the restricted scope of attack scenarios. Furthermore, in general, datasets consider different attack types and features, which become even more challenging for the generalization of the results. These factors could constrain the direct generalization of our findings.

Nevertheless, the dataset provides a rare and valuable testbed that integrates both biomedical sensor readings and network traffic features, aligning well with the patient-centric vision of Healthcare 5.0, as shown previously in Table 2. Our results, therefore, demonstrate the potential of explainable IDS models to simultaneously address cybersecurity threats and clinical interpretability needs. While the absolute performance metrics may vary in larger or more heterogeneous deployments, the methodological insights—such as the integration of network and physiological features, and the use of explainable models—are transferable to other Healthcare 5.0 contexts. Future research should expand on these findings by validating IDS models on broader datasets collected from real-world clinical environments.

The next section discusses these open issues, outlining challenges that must be addressed to advance secure and reliable healthcare systems.

## 7. Open Issues

Despite substantial advancements in the design and implementation of IDSs for Healthcare 5.0, numerous unsolved challenges continue to hinder their widespread efficacy and deployment. Addressing these issues is imperative for ensuring the development of efficient security frameworks capable of safeguarding the integrity, confidentiality, and availability of next-generation healthcare systems.

### 7.1. Data-Related Challenges

Healthcare 5.0 environments are characterized by massive, heterogeneous, and sensitive data streams. Current IDS frameworks face limitations in handling:

- Dataset Quality: Building robust, high-quality datasets that accurately represent IoMT attack scenarios is essential for the development of effective IDSs [13,14,21]. However, these datasets are often highly dimensional, containing numerous features. This leads to the curse of dimensionality, which makes it challenging to identify relevant features and understand their contribution to model outputs [23,24].
- Data Scarcity and Heterogeneity: The development of effective IDSs in healthcare is hindered by the scarcity and heterogeneity of available datasets. Few publicly available IoMT-specific datasets exist, limiting the evaluation of IDS performance in realistic settings. Existing datasets often lack realism and diversity, and most are siloed—focusing on either clinical or network data independently [13,14,37]. Integrating both domains could enrich contextual information, but simultaneously raises concerns of expanded attack surfaces and patient privacy. In addition, the lack of comprehensive datasets in terms of, e.g., considered attacks and features, constitutes a challenge for the generalization of research findings. Recently, GANs have been proposed to synthesize realistic, privacy-preserving data for IDS training and to mitigate imbalance in healthcare datasets [91,92]. While promising, these approaches require careful design to ensure that synthetic data improves detection without compromising patient confidentiality. Future research must explicitly address this privacy–utility trade-off.
- Data Privacy and Confidentiality: It remains a fundamental challenge to ensure the secure handling of IoMT data, particularly within highly regulated environments [12]. Although generative models like GANs provide a promising method for producing realistic synthetic data, they raise important concerns regarding the trade-offs between fidelity (how well synthetic data replicates real data), utility (its effectiveness for downstream tasks), and privacy [93]. Beyond GANs, recent approaches have explored instruction-tuned LLMs (Large Language Models) to capture inter-row relationships and mitigate memorization risks, in which metrics like DLT (Distance-Based Leakage Test) and LLE (Local Leakage Estimation) show improved privacy protection without compromising classification performance [94]. However, such models still face limitations, including high computational cost, limited support for regression tasks, and a lack of formal Differential Privacy (DP) guarantees [95], in addition to risks of algorithmic bias [96]. More recently, score-based diffusion in a VAE-learned (Variational Autoencoder) latent space has been applied to handle mixed-type tabular data, improving generation quality and sampling speed [97]. Yet, this latent-diffusion approach introduces new questions regarding the interpretability and robustness of latent representations, scalability, and compatibility with privacy mechanisms. Recent frameworks show that combining FL with edge computing can preserve privacy while still enabling collaborative IoMT analytics [98].

### 7.2. Model-Related Challenges

IDS models for Healthcare 5.0 must be both accurate and trustworthy. However, they face significant barriers, including the following:

- Enhanced Data Understanding: Improving feature correlation understanding to reduce data dimensionality and enhance model performance by filtering redundant or irrelevant features [12,21]. Beyond traditional feature selection algorithms, XAI has been presented as a prominent direction toward dimensionality reduction and model performance enhancing [99,100].
- Adversarial Robustness: Adversarial examples are deliberately perturbed inputs designed to mislead ML models, often through modifications that are imperceptible to humans. IDSs are particularly susceptible to such attacks, which can result in the misclassification of malicious traffic as benign and consequently compromise network security. Although recent advancements have improved the robustness against adversarial examples, existing approaches remain limited, particularly against diverse and black-box adversarial strategies. Enhancing robustness across heterogeneous ML/DL architectures may rely on adversarial training strategies that combine attack sample generation, robust preprocessing, and GAN-based defenses [101].
- XAI-Driven Adversarial Attacks: In XAI-driven attacks, adversaries take advantage of the insights provided by XAI techniques to identify which input features influence the model’s decisions the most, allowing them to craft more targeted and effective adversarial examples [102]. Recent works [103,104] have shown that such attacks can succeed even in black-box settings, where the attacker has no access to the internals of the model. These techniques pose a significant threat to model reliability and trust, particularly in sensitive fields such as cybersecurity and healthcare. Mitigating this risk requires the development of defense strategies capable of withstanding adversarial inputs informed by model explanations.
- Explainability and Transparency: Evaluating the quality and reliability of XAI-generated explanations is challenging and requires standardized benchmarks to objectively assess their fidelity, completeness, and usefulness. Additionally, explanations must be understandable and actionable for both experts and non-experts, necessitating intuitive, user-centered interfaces to ensure effective comprehension and adoption [5,16,23].
- Model Explainability Trade-Off: The balance between the complexity of advanced AI models and the need for interpretability remains a critical challenge in healthcare, as highly accurate models often sacrifice transparency, potentially lowering trust between healthcare professionals [22,23]. Additionally, incorporating XAI into IDSs, while improving transparency, also risks exposing proprietary model details, potentially leading to intellectual property loss or adversarial attacks [22].
- Automated Solutions: A significant gap remains in developing reliable, automated, and user-friendly XAI solutions capable of delivering clear and trustworthy explanations for clinical decision support [24].

### 7.3. Deployment and Operational Challenges

Deploying IDSs in real-world Healthcare 5.0 settings introduces additional challenges:

- Addressing AI Model and Infrastructure Challenges: Tackling issues arising from the distributed nature of IoMT devices, large data volumes, hardware constraints, and evolving data environments [21], alongside advancing the standardization of healthcare IT infrastructure to enhance security, interoperability, and the performance of AI-based systems [13]. Emerging paradigms such as FL and edge computing have shown promise in mitigating data scarcity and privacy concerns by enabling collaborative, privacy-preserving model training while offloading computation from resource-constrained IoMT devices [98].
- Real-Time Data Management: Efficiently handling large-scale, real-time data streams remains a critical challenge, particularly in balancing security with minimal performance overhead on constrained devices [13,14,21,35]. While XAI techniques can be computationally demanding, recent studies highlight the need for efficient and scalable explainability models [24], federated edge processing for real-time monitoring [105], and decentralized explainable intrusion detection frameworks [106]. Furthermore, comprehensive reviews emphasize the integration of explainable AI with federated learning as a key pathway for enabling trustworthy and scalable next-generation IoT systems [107]. Nevertheless, achieving dependable and secure performance under sudden surges in patient data or rapid device proliferation remains an open research problem.
- Zero-Day Exploits: The rise in sophisticated cyber threats in Healthcare 5.0, such as zero-day exploits and ransomware targeting IoMT devices [21], threatens patient safety and data integrity. This demands IDSs capable of adapting to evolving threats within the dynamic IoMT ecosystem.
- Integration of Emerging Technologies: Leveraging emerging technologies such as 5G, AI, ML, and blockchain for enhancing the security, reliability, and efficiency of IoMT systems. This includes developing secure network slicing and energy-efficient protocols to support the growing demands of healthcare applications. Additionally, integrating edge and fog computing enables the deployment of distributed and resource-efficient IDS architectures, which help reduce latency and optimize resource consumption while maintaining robust security. The literature indicates that both in edge and fog scenarios, cryptography consists of an important mechanism to reach patient data privacy-preserving [12,21].
- Scalability, Flexibility, and Efficient Resource Management: Creating scalable IDS frameworks that can accommodate the heterogeneous and expanding IoMT device ecosystem [12,21].
- Secure Data Sharing and Interoperability: Establishing standards for secure, seamless data exchange across different healthcare platforms and devices [12].

### 7.4. Regulatory, Ethical, and Compliance Issues

Ensuring compliance with healthcare regulations while maintaining effective intrusion detection remains a significant challenge in the IoMT landscape [15]. Organizations must navigate complex regulatory frameworks while protecting sensitive patient data and ensuring system integrity. This necessitates the development of standardized security measures and communication protocols specifically tailored to IoMT environments, which are crucial for achieving reliable intrusion detection and prevention while upholding ethical and legal obligations [12,21].

Having identified the key open issues in IDSs for Healthcare 5.0, the final section summarizes the main findings of this survey and reflects on the implications for secure, explainable, and patient-centered healthcare systems.

## 8. Conclusions

This survey has provided an overview of IDSs tailored for Healthcare 5.0—framing security not as an isolated concern, but as a core component of human–cyber–physical collaboration. It traces the evolution of healthcare paradigms over time, emphasizing the shift from the device-centric architectures of Healthcare 4.0 to the patient-oriented vision of Healthcare 5.0. Both from theoretical and practical perspectives, we demonstrate how AI-driven IDSs must intertwine with clinical workflows and sensitive biomedical data. Furthermore, we highlight the dual role of explainability—not only as a means to enhance transparency and trust, but also as a critical element within a broader socio-technical security landscape. At the same time, we identify a gap in the application of XAI to IDSs in Healthcare 5.0, underscoring the need for intensive, interdisciplinary efforts to develop interpretable solutions that serve patients, clinicians, researchers, and other stakeholders in the healthcare ecosystem.

One critical limitation identified is the absence of publicly available and robust, high-quality Healthcare 5.0-specific datasets, as illustrated in Table 2. Current research often relies on generalized or outdated datasets, which do not reflect IoMT device behaviors, traffic patterns, or attack vectors unique to modern healthcare environments. This gap hinders the development, validation, and benchmarking of IDS solutions tailored to Healthcare 5.0. Furthermore, our study has identified a lack of incorporation of biomedical data within datasets that could make the model predictions and decisions more human-centric.

Future work should concentrate on creating standardized, representative datasets and lightweight IDS models suitable for resource-constrained IoMT devices. FL approaches may offer a secure, collaborative model for training across distributed healthcare networks. Establishing common evaluation metrics will facilitate fair comparison of emerging methods. Addressing these issues is essential for deploying robust, explainable, and efficient IDSs that can reliably protect next-generation healthcare systems.

## Figures and Tables

**Table 1:** Related surveys on IDSs, XAI, and Healthcare 5.0.

Section	Reference	Healthcare 5.0	IDS	Biomedical Data	XAI	Practical Case Study
(Section 3.2)	[12]	✓	✓	✗	✗	✗
	[13]	✓	✓	✗	✗	✗
	[14]	✓	✓	✗	✗	✗
	[15]	✓	✓	✗	✗	✗
	[16]	✓	✓	✗	✗	✗
	[17]	✓	✓	✗	✗	✗
	[18]	✓	✓	✗	✗	✗
	[19]	✓	✓	✗	✗	✗
	[20]	✓	✓	✗	✗	✗
	[21]	✓	✓	✗	✗	✗
(Section 3.3)	[22]	✓	✗	✓	✓	✗
	[23]	✓	✗	✓	✓	✗
	[24]	✓	✗	✓	✓	✗
	[25]	✓	✗	✓	✓	✗
	[5]	✓	✗	✓	✓	✓
	Our Survey	✓	✓	✓	✓	✓

**Table 2:** Healthcare IDS datasets overview.

Dataset	Biomedical		IoT Devices	Network Data	Data Source	Availability	Healthcare 5.0 Alignment
	Sensor	Data					
CICIoMT2024 [80]	Yes	No	IIoMT	Yes	TestBed	Public	Partial
WUSTL-HDRL-2024 [81]	No	No	IoMT 5G	Yes	Emulated	Public	Partial
WUSTL-EHMS-2020 [37]	Yes	Yes	IoMT	Yes	TestBed	Public	Strong
ECU-IoHT [84]	Yes	No	IoHT	Yes	TestBed	Public	Partial
BlueTack [85]	Yes	No	IIoMT	Yes	TestBed	Public	Partial
ICU (IoT-Flock) [83]	Yes	No	IoMT	Yes	Emulated	Public	Weak
IoMT-TrafficData [36]	Yes	No	IoMT	Yes	TestBed	Public	Partial
LDE/CDE [86]	Yes	No	IoMT	Yes	TestBed	Unavailable	Weak

**Table 3:** List of features from the WUSTL-EHMS-2020 dataset and their preprocessing status.

#	Feature	Type	Description	Status
**Network Flow Features**				
1	SrcAddr	Categorical	Source Address	Removed
2	DstAddr	Categorical	Destination Address	Removed
3	Sport	Integer	Source Port	Converted
4	Dport	Integer	Destination Port	Converted
5	SrcBytes	Integer	Source Bytes	Retained
6	DstBytes	Integer	Destination Bytes	Retained
7	SrcLoad	Float	Source Load	Retained
8	DstLoad	Float	Destination Load	Retained
9	SrcGap	Integer	Source Missing Bytes	Retained
10	DstGap	Integer	Destination Missing Bytes	Retained
11	SIntPkt	Float	Source Inter Packet Time	Retained
12	DIntPkt	Float	Destination Inter Packet Time	Retained
13	SIntPktAct	Float	Source Active Inter Packet Time	Retained
14	DIntPktAct	Integer	Destination Active Inter Packet Time	Retained
15	SrcJitter	Float	Source Jitter	Retained
16	DstJitter	Float	Destination Jitter	Retained
17	sMaxPktSz	Integer	Source Max Packet Size	Retained
18	dMaxPktSz	Integer	Destination Max Packet Size	Retained
19	sMinPktSz	Integer	Source Min Packet Size	Retained
20	dMinPktSz	Integer	Destination Min Packet Size	Retained
21	Dur	Float	Duration of Flow	Retained
22	Trans	Integer	Aggregated Packet Count	Retained
23	TotPkts	Integer	Total Packet Count	Retained
24	TotBytes	Integer	Total Byte Count	Retained
25	Load	Float	Average Load	Retained
26	Loss	Integer	Dropped or Retransmitted Packets	Retained
27	pLoss	Float	Packet Loss Rate	Retained
28	pSrcLoss	Float	Source Packet Loss Rate	Retained
29	pDstLoss	Float	Destination Packet Loss Rate	Retained
30	Rate	Float	Packets per Second	Retained
31	DstMac	Categorical	Destination MAC Address	Removed
32	Dir	Categorical	Unknown Direction	Removed
33	Flgs	Categorical	Unknown Flags	Removed
34	SrcMac	Categorical	Source MAC Address	Removed
35	Packet_num	Integer	Packet Number	Removed
**Biometric Features**				
36	Temp	Float	Patient Temperature	Retained
37	SpO2	Integer	Peripheral Oxygen Saturation	Retained
38	Pulse_Rate	Integer	Pulse Rate	Retained
39	SYS	Integer	Systolic Blood Pressure	Retained
40	DIA	Integer	Diastolic Blood Pressure	Retained
41	Heart_Rate	Integer	Heart Rate	Retained
42	Resp_Rate	Integer	Respiration Rate	Retained
43	ST	Float	ECG ST Segment	Retained
**Target Variable**				
44	Category Label	Categorical	Multiclass label (e.g., Normal, Spoofing, Data Injection)	Converted
45	Label	Integer	Binary Attack Indicator (0: Benign, 1: Attack)	Removed

## Data Availability

The data supporting the experimental case study are publicly available as part of the WUSTL-EHMS-2020 dataset, available as of 2 February 2025. https://www.cse.wustl.edu/jain/ehms/index.html. No new data were generated in this study.
